# Seven-Year Visual and Anatomical Outcomes of Intravitreal Vascular Endothelial Growth Factor Inhibition for Neovascular Age-Related Macular Degeneration

**DOI:** 10.1155/2020/8345850

**Published:** 2020-02-14

**Authors:** Martin Stattin, Julia Forster, Daniel Ahmed, Anna-Maria Haas, Alexandra Graf, Katharina Krepler, Siamak Ansari-Shahrezaei

**Affiliations:** ^1^Karl Landsteiner Institute for Retinal Research and Imaging, Vienna, Austria; ^2^Department of Ophthalmology, Rudolf Foundation Hospital, Juchgasse 25, Vienna 1030, Austria; ^3^Center for Medical Statistics, Informatics, and Intelligent Systems, Medical University of Vienna, Spitalgasse 23, Vienna 1090, Austria; ^4^Department of Ophthalmology, Medical University of Graz, Auenbruggerplatz 1, Graz 8036, Austria

## Abstract

**Purpose:**

To evaluate 7-year visual and anatomical outcomes of intravitreal injections (IVI) with antivascular endothelial growth factor (anti-VEGF) for neovascular age-related macular degeneration (nAMD) based on a personalized pro re nata (PRN) regimen.

**Methods:**

Anonymized data of 124 consecutive eyes in 121 patients with treatment-naïve nAMD were initially collected in 2010. Of those, 45 received anti-VEGF IVI at least every 6months until 2017 in one single center in Austria and hence were retrospectively analyzed. All eyes had been initiated on a loading dose of 3 monthly IVI with different anti-VEGF agents followed by a PRN regimen in the first year. At year 2, monitoring as well as therapeutic intervention could be prolonged every 2weeks up to intervals of 3months without capping treatment. Primary outcome measure was the change of visual acuity (VA) assessed by Early Treatment Diabetic Retinopathy Study charts at 4 meters (ETDRS) in letters—counting every correctly read letter—and converted to Snellen. Secondary outcome measures were number of injections and change of central retinal thickness (CMT) from baseline.

**Results:**

Mean baseline VA was 20/63 + 1 (0.63 ± 0.26 ETDRS) and declined to 20/100 + 2 (0.45 ± 0.33) with an overall loss of 9 letters ETDRS after 7years (*p* = 0.001). An average of 3.5 ± 1.9 IVI was given per year and eye. Mean CMT at baseline was 322 ± 95 *μ*m, decreased by 52 *μ*m, decreased by 52 *μ*m, decreased by 52 *μ*m, decreased by 52 *μ*m to 270 ± 70 *μ*m within the first year, and remained below baseline at year 7 (271 ± 106 *μ*m; *p* = 0.001). An average of 3.5 ± 1.9 IVI was given per year and eye. Mean CMT at baseline was 322 ± 95 *μ*m, decreased by 52 *μ*m to 270 ± 70 *μ*m within the first year, and remained below baseline at year 7 (271 ± 106 *μ*m; *p* < 0.001).

**Conclusions:**

Our data confirm an absolute vision loss in eyes compromised by nAMD after 7 years of continuous VEGF inhibition. The visual decline was significantly related to baseline VA as well as the number of injections. We suggest following patients thoroughly independent of the initial VA and a greater incentive for the physician to treat.

## 1. Introduction

Late-stage age-related macular degeneration (AMD) is a substantial burden for patients and doctors in developed countries [1]. The projected number of patients with age-related macular degeneration in 2020 is 196 million, increasing to 288 million in 2040 [2]. Its neovascular entity accounts for only 10–20% of cases but is responsible for 80–90% of severe visual loss and progresses rapidly if left untreated [3, 4].

Large multicenter clinical trials have proven the efficacy of monthly intravitreal antivascular endothelial growth factor (anti-VEGF) therapy in treating neovascular (*n*)AMD for at least 2years [5, 6]. New drugs with prolonged injection intervals have become available in the past years [7]. Considering the excessive costs for health care systems, restricted capacity in clinical practice and risk for patients, alternative approaches like treatment as needed (pro re nata; PRN), or certain retreatment while extending intervals (treat and extend; TAE) have been explored [8–11]. Short-term clinical trials have shown similar visual outcomes for all applied strategies [12–17]. A few prolonged studies have been published in the past years with varying results [18–20]. Real-world long-term data of different treatment strategies are needed to reflect maintenance of efficacy and safety over time.

In the light of the above, we analyzed 7year visual and anatomical outcomes of a personalized PRN treatment regimen with anti-VEGF for nAMD from one treatment center in Austria.

## 2. Materials and Methods

This was a retrospective, observational, cross-sectional data analysis. The study protocol adhered to the tenets of the Declaration of Helsinki.

### 2.1. Patients

Data of 127 eyes with treatment-naïve nAMD in 124 patients were initially collected in a consecutive manner. Of those, 124 eyes were eligible for enrollment. Forty-five eyes in 45 patients who commenced therapy with intravitreal anti-VEGF in 2010 at our tertiary eye care center (Medical Retina Unit, Department of Ophthalmology, Rudolf Foundation Hospital, Vienna) and who received intravitreal injections (IVI) for at least 7years could be analyzed. Patients with injection-free intervals of more than 6 months were excluded from analysis. Cataract surgery during follow-up was not an exclusion criterion.

### 2.2. Baseline Assessment

All patients underwent a complete ophthalmic examination including best-corrected visual acuity (BCVA) using the Early Treatment Diabetic Retinopathy Study charts at 4 meters (ETDRS)—counting every correctly read letter—as well as by indirect slit-lamp biomicroscopy (Haag-Streit AG, Switzerland) with dilated pupils using 0.5% tropicamide (Mydriaticum®, Agepha Pharmaceuticals, Vienna, Austria) and 2.5% phenylephrine drops. The diagnosis of nAMD was confirmed by dye-based angiography (Spectralis HRA-OCT Confocal Scanning Laser Ophthalmoscope and Angiography; Heidelberg Engineering, Heidelberg, Germany). The follow-up and further procedures regarding decision-making were regularly based on BCVA, slit-lamp biomicroscopy, and spectral domain-optical coherence tomography (SD-OCT; Cirrus HD 4000, Carl Zeiss Meditec AG, Germany). The central macular thickness (CMT) was measured by a SD-OCT B-scan within the central 1 mm zone.

### 2.3. Treatment Protocol and Follow-Up

**Figure 1:**
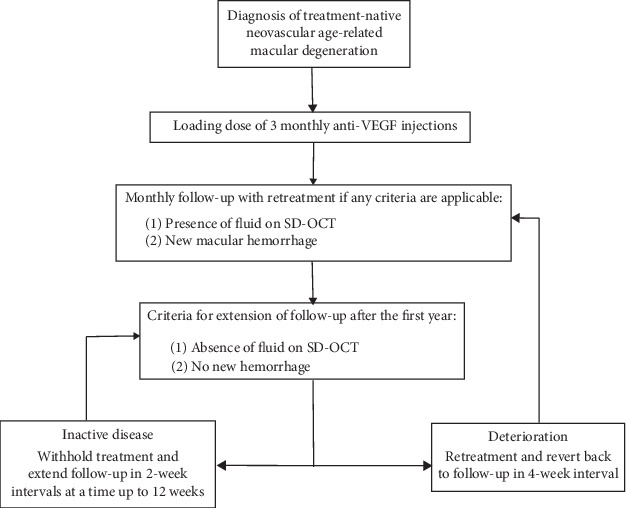
Personalized pro re nata treatment algorithm.

Consenting patients received three consecutive monthly intravitreal anti-VEGF injections with different agents (aflibercept 2 mg, bevacizumab 1.25 mg, ranibizumab 0.5 mg). All patients continued on a PRN regimen with monthly visits and injections as needed within 5 working days in the first year. A medical retina fellow or senior evaluated the further treatment at each follow-up based on previously established criteria (Figure 1).

After year 1, monitoring visits were extended every 2weeks to a maximum of 3 months if the disease was inactive, but injections could be withheld for longer periods without capping. We considered 6 months of inactivity as stable disease and as a consequence excluded the eye from analysis. In other words, eyes had to be treated at least twice a year to be eligible for participation. Switching the anti-VEGF agent was left to the surveilling ophthalmologist but did not change treatment intervals. The occurrence of systemic cerebrovascular, cardiovascular, and ocular adverse events (AE) was documented and did not necessarily postpone the anti-VEGF treatment.

### 2.4. Data and Statistical Analysis

Patient charts were reviewed for BCVA, CMT, numbers of IVI, and the incidence of AE. The analysis was performed using SAS version 9.4 (SAS Institute Cary NC, USA), Microsoft Excel 2007 (12.0.4518.1014), and R release 3.2.1. Univariate regression models were calculated to investigate the influence of time, eye, age at baseline, the average number of IVI, and the occurrence of AE on main outcome measures. All potential influencing significant factors in the univariate analysis were re-evaluated in a multivariable model. Continuous baseline variables were compared between eyes with 7 years of observation period using ANOVA. Categorical (laterality, sex, AE, and time) baseline variables were compared using Chi-squared tests. A *p* value <0.05 was rated statistically significant.

## 3. Results

**Figure 2:**
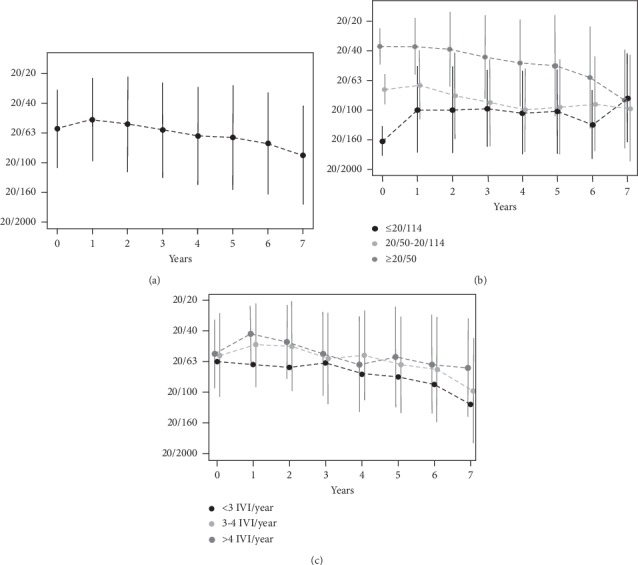
Mean visual acuity (VA) measured by Early Treatment Diabetic Retinopathy Study (ETDRS) charts at 4 meters and converted to Snellen over 7 years. (a) Mean change of VA for all eyes. (b) Mean change of VA separated in eyes with good baseline VA ≥ 20/50 (0.7 ETDRS), intermediate VA 20/50–20/114 (0.7–0.35 ETDRS), and minor VA ≤ 20/114 (0.35 ETDRS). (c) Mean change of VA subdivided into three groups based on the average numbers of intravitreal injections per year.

The mean patient's age at initial presentation was 76 ± 7.5 years with a female preponderance (77%) and an evenly distributed laterality. Overall, baseline mean VA was 20/63 + 1 (0.63 ± 0.26 ETDRS). Forty-five (37%) eyes with nAMD were eligible for enrollment after 7 years. The baseline data of this population were comparable with a slightly higher age (77.7 ± 6.1 years), also a female dominance (85%) but more right eyes (57%), while the initial mean VA was 20/63 + 3 (0.66 ± 0.24 ETDRS). In total, mean VA raised significantly within the first year to 20/50–1 (0.69 ± 0.28 ETDRS; *p*=0.044 (95% CI: 0.001; 0.09)). No difference was found between baseline mean VA and the second or the third year. A significant mean visual loss became evident in the following years 4–6. Mean VA declined to 20/100 + 2 (0.45 ± 0.33 ETDRS) in year 7 (Table 1; Figure 2(a); *p* < 0.001 (95% CI: −0.23; −0.11)).

In a subanalysis, 65 of 124 (52%) eyes had a VA ≥ 20/50 (0.7 ETDRS) at the baseline, 37 of 124 (30%) eyes had an intermediate VA between 20/50 (0.7 ETDRS) and 20/114 (0.35 ETDRS), while 22 of 124 (18%) eyes had an initial VA ≤ 20/114 (0.35 ETDRS) (Figure 2(b); *p* < 0.001 (95% CI: −0.23; −0.11)). Regarding the VA at 7 years, comparable relative numbers in 25 of 45 (56%) eyes with good initial VA lost 18 letters, 15 of 45 (33%) eyes with intermediate VA lost 6 letters, while 5 of 45 (11%) eyes with minor initial VA gained 14 letters. A significant difference between good VA and minor VA at baseline (*p* < 0.001 (95% CI: −0.5; −0.28)) as well as intermediate VA and good VA at baseline could be detected after 7years (Figure 2(b); *p* < 0.001 (95% CI: −0.36; −0.19)). Overall, 3.5 ± 1.9 IVI per eye and year was given. The number of IVI administered over time was significantly related to the outcome of VA (*p*=0.011 (95% CI: 0.01; 0.05)). For further analysis, the eyes were subdivided into 3 groups (<3IVI/year; 3–4 IVI/year; >4 IVI/year) based on the average number of IVI given per year. Eyes with a follow-up of 7 years had more IVI/year (4.0 ± 2.0) than the average number administered in total. After 7 years, 13 of 45 (29%) eyes with less than 3 IVI/year lost 19 letters on an average, and 12 of 45 (27%) eyes with 3 to 4 IVI/year lost 11 letters, while 20 of 45 (44%) eyes treated with more than 4 IVI/year lost 4 letters (Figure 2(c)). Neither age nor sex, laterality nor the occurrence of adverse events (AE) had an impact on VA.

**Figure 3:**
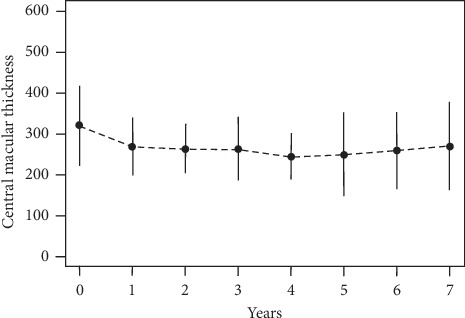
Mean change of central macular thickness for all eyes measured by spectral domain-optical coherence tomography B-scans over time.

The mean CMT was 322 ± 95 *μ*m at the baseline. For the 7-year subgroup, a comparable CMT of 325 ± 59 *μ*m was evident. A significant thinning compared with the baseline was measured at all time points (*p* < 0.001 (95% CI: −83.84; −33.90)). A reduction of 52 *μ*m became apparent within the first year, declined to 246 ± 56 *μ*m after 4years, and remained at 271 ± 106 *μ*m on average in year 7 (Figure 3).

Neither VA at baseline nor age, sex, laterality, nor the number of IVI was significantly related to a change in CMT (*p*=0.308).

Severe ocular events AE were documented in 2 (1.6%) eyes (retinal tear, elevated IOP >30 mmHg), and no cases of endophthalmitis were reported. Nine patients had an episode of stroke or myocardial infarction during the observation period. These eyes were not included in the 7-year data analysis.

## 4. Discussion

In this retrospective data analysis, we evaluated a representative number of eyes complicated by nAMD and treated with a personalized PRN regimen at least every 6 months for 7 years. A visual decline of nearly 2 lines of ETDRS became evident with a low number of 3.5 IVI/year based on our protocol, which relies on empiric variable intervals for each patient and was published in 2019 [21]. It mixes advantages of both, PRN and TAE, as it combines a fairly low injection rate with an acceptable clinical effort, a limited risk besides minimum undertreatment.

A few studies on long-term anti-VEGF treatment for nAMD have been published yet. Gillies et al. extracted heterogeneous data of 1212 eyes from a multicentered registry, of which 131 were being followed for 7 years [22]. The multicenter SEVEN-UP study reported on 65 patients treated with intravitreal ranibizumab and different regimens for a mean of 7.3years with a decline of 8.6 letters [19]. Patients were treated either monthly or PRN for 24 months before entering a quarterly PRN protocol for another 2 years. An average of 6.8 IVI was given in the last 3.4 years after the exit from HARBOR protocol, resulting in 1.6 IVI/year and study eye. This low number was partially attributed to the study design. The authors did not exclude eyes without treatment in the last years, while our cohort reflects only eyes with the need of at least 2 injections per year. It seems difficult to acquire representative data of a homogeneous cohort for an interpretation over an extended time period. In our study, a uniform data collection was possible with the help of continuous observation and minimum diversity in respect of the medical staff including 2 medical retina specialists as well as a single center setting in an urban environment. Our low number of average IVI/year is most likely related to a compensation for a higher number of injections in the first years and a low number due to less burden for treatment in the latter years.

Although an absolute visual loss became evident in the total number of eyes, their separation based on baseline VA led to interesting findings: the better BCVA at baseline, the more letters were lost after 7years. Our results were concordant with those of other authors as well as the UK Age-Related Macular Degeneration EMR Users Group, who previously described similar effects [23, 24]. “Ceiling” is referred to as the limited potential gain in vision simply due to a relatively good BCVA at the baseline. The terminus “floor” was used in initially poor vision where loosing was unlikely but gaining an option. It looks like this phenomenon was true for all eyes compromised by nAMD with long-term treatment, independent of the underlying modality.

In general, 3 different regimens have been established in the past years. Fixed continuous dosing could rarely be prolonged and was never practical because of certain overtreatment besides cost and burden to patients as well as clinicians. Reality led to an interest in different therapeutic approaches with encouraging visual results in short-term studies [12, 25]. PRN with fixed visits and variable injection intervals based on the disease activity included the advantages of fewer burden to the patient with more cost-effective management in the long-term [26]. TAE was introduced as injection and extended by Spaide in 2007 and was based on the strategy of minimizing recurrences by retreating even without signs of activity while expanding the intervals [11, 27]. This protocol reduced the number of visits and tests but increased the potential of overtreatment. Lately, it was widely adopted in centers across the U.S. Considerably good 8-year TAE outcomes were published recently by a Scandinavian group [20]. Nevertheless, noninferior VA could only be achieved by a relatively large number of IVI. We also investigated the effect of the numbers of injections given per year and found significant differences. A fair loss of 1-line ETDRS in 7 years could be established with only 1 more injection on average per year. Considering the rising number of elderly patients affected by this chronic disease as well as the associated financial and social burden, it should be our primary goal to limit the number of IVI/year but at the same time to sustain an acceptable visual outcome for our patients to conquer their daily routine.

CMT measurements were collected as a secondary outcome. The significant macular thinning in OCT B-scans within the first year has been proposed by various 1-year registration trials [8–11]. In our study cohort, the reduction at year 1 could be preserved throughout the 7-year observation period, independent of the number of IVI.

This paper has several limitations. Its retrospective design questions many variables likely to be evaluated in a prospective study. No endpoints at predetermined time intervals were set. Eyes submitted to cataract surgery were included and likely to demonstrate considerably higher BCVA. Recent data from the CATT Research Group, who investigated the development of geographic atrophy (GA) in eyes complicated by nAMD, showed an incidence of 38% among these 5 years after initiating therapy [28]. Potential GA as a source of the visual decline was not assessed in our study. The three predominantly administered intravitreal medications were exchanged randomly if eyes seemed to respond inadequately. The switch was not investigated separately due to the retrospective data collection. Thus, our findings reflect real-life outcomes. This study's strength is its long observation period and data recording by means of well-established repeatable methods and personnel for a reasonably large number of eyes.

## 5. Conclusions

In conclusion, our real-world data confirm an absolute visual loss of 9 ETDRS letters with a reasonably low number of injections per year enabled by a personalized PRN regimen over a time period of 7 years. The response to treatment needs to be addressed thoroughly regardless of chronicity or baseline VA. Retreatment should be considered in doubt to largely avoid undertreatment. The initial submacular thinning in the first year could be preserved over time. Many questions are still to be answered in a prospective manner. Reliable data of long-term treatment effects following VEGF inhibition are mandatory to conquer this disease in the future.

## Figures and Tables

**Table 1:** Mean VA development for the respective cohort from baseline to each year in total, separated in baseline VA and amount of intravitreal injections/year in Snellen.

Years	VA (*n*)	≥20/50 (*n*)	20/50–20/114 (*n*)	≤20/114 (*n*)	<3 IVI/year (*n*)	34 IVI/year (*n*)	>4 IVI/year (*n*)

0	20/63 + 1 (124)	20/40 + 1 (65)	20/80 + 2 (37)	20/160–1 (22)	20/63 (50)	20/63 + 2 (31)	20/63 + 2 (43)
1	20/50 + 1 (119)	20/40 + 1 (62)	20/63–1 (33)	20/100 (20)	20/63–1 (43)	20/50 (31)	20/40–2 (44)
2	20/50–2 (119)	20/40 (64)	20/80 (32)	20/100 (20)	20/63–2 (43)	20/50 (32)	20/50 + 1 (44)
3	20/63 + 1 (119)	20/40–2 (64)	20/100 + 2 (32)	20/100 (20)	20/63–1 (42)	20/63 + 1 (33)	20/63 + 2 (44)
4	20/63–1 (119)	20/50 + 1 (64)	20/100 (33)	20/100–1 (19)	20/80 + 1 (42)	20/63 + 2 (33)	20/63–1 (44)
5	20/63–1 (119)	20/50 (63)	20/100 + 1 (34)	20/100–1 (20)	20/80 (42)	20/63–1 (33)	20/63 + 1 (44)
6	20/80 + 1 (87)	20/63 + 1 (51)	20/100 + 2 (22)	20/125 (13)	20/100 + 2 (32)	20/80 + 2 (23)	20/63–1 (32)
7	20/100 + 2 (45)	20/80–2 (25)	20/100 (15)	20/80–1 (5)	20/125 + 1 (13)	20/100 (12)	20/63–2 (20)
Letters^*∗*^	−9	−18	−6	+14	−19	−11	−4

VA = visual acuity; *n* = number of eyes; IVI = intravitreal injection; ^*∗*^change in letters ETDRS = Early treatment diabetic retinopathy study chart at 4 meters.

## Data Availability

The data used to support the findings of this study are available from the corresponding author upon request.
